# Coping With the COVID-19 Pandemic: Examining Gender Differences in Stress and Mental Health Among University Students

**DOI:** 10.3389/fpsyt.2021.650759

**Published:** 2021-04-07

**Authors:** Rebecca Prowse, Frances Sherratt, Alfonso Abizaid, Robert L. Gabrys, Kim G. C. Hellemans, Zachary R. Patterson, Robyn J. McQuaid

**Affiliations:** ^1^Department of Neuroscience, Carleton University, Ottawa, ON, Canada; ^2^University of Ottawa Institute of Mental Health Research, Ottawa, ON, Canada; ^3^School of Psychology, University of Ottawa, Ottawa, ON, Canada

**Keywords:** coping, COVID-19, emerging adults, mental health, university students, stress

## Abstract

The COVID-19 pandemic has imposed a wide variety of unprecedented challenges, many of which appear to be disproportionately affecting the mental health and well-being of young adults. While there is evidence to suggest university students experience high rates of mental health disorders, less is known about the specific impacts of the COVID-19 pandemic on student mental health and how they are coping with this stress. To address this gap, we conducted an online study among undergraduate students (*n* = 366) to examine the impact of the COVID-19 pandemic on academics, social isolation, and mental health, as well as the extent to which they have been implementing a variety of coping strategies. The pandemic had a more pronounced negative effect on female students' academics, social isolation, stress and mental health compared to male counterparts. Moreover, for females, frequent use of social media as a coping mechanism was associated with greater perceived negative impacts on their academic performance and stress levels, compared to males. However, frequent social media use related to similar negative mental health effects for both males and females. While male and female students both reported using substances to cope, for males the use of cannabis was associated with greater negative impacts on academic outcomes, stress and mental health compared to females. These findings highlight the need for adequate student support services across the post-secondary sector, and point to the importance of gender informed interventions to address the impacts of the COVID-19 pandemic.

## Introduction

The COVID-19 pandemic has introduced a unique set of challenges and stressors that have negatively impacted mental health and wellness (1–3). In addition, specific sociodemographic groups are being disproportionately affected. In this regard, emerging data indicate that the pandemic is negatively influencing the mental health and increased self-reports of loneliness in younger populations more so than in any other age group (4–6). This might be due, in part, to young individuals' educational, economic, and social lives being highly disrupted by the public health crisis (7). For example, higher levels of depression and loneliness have been reported among adolescents and young adults, which has been attributable to the increased stress associated with the pandemic (8–10). Recent data also indicate that females are at increased risk of loneliness, depression and anxiety during COVID-19 (11–14).

University students represent one group of young or emerging adults (aged 18–25) that have been particularly impacted by COVID-19 (15). With the quick closures of universities, students encountered uncertainty and concern about their academic future, as well as social isolation and a lack of supports (16). High levels of stress, anxiety and depression are prevalent among post-secondary education students (17), in part because of the academic, social, and personal demands of navigating through higher education (18). More generally, emerging adulthood is considered a vulnerable period that coincides with the onset of mental health disorders, such as anxiety and depression (19), which can negatively impact developmental trajectories by reducing academic achievements, increasing substance use and poor health behaviors (20). Indeed, even prior to the COVID-19 pandemic, high rates of mental health concerns existed with ~35% of post-secondary students reporting a lifetime mental health disorder (21). University students also reported high rates of substance use, with 62.8% reporting alcohol use and 24.7% reporting cannabis use in the past month (17), potentially as a method of coping with the stress they experience as students (22, 23). Thus, the COVID-19 pandemic may exacerbate these issues, occurring at an already vulnerable time period, as university students suffer the psychological impacts of the COVID-19 lockdown (8, 16). Preliminary data indicate that the impact of remote learning may have negative consequences on student well-being, as a recent study reported higher levels of stress and isolation as well as negative mood during a synchronous online learning experience, compared to a traditional face-to-face learning environment (24).

While it is evident that the COVID-19 pandemic is having a significant negative impact on students, the full range and nature of its impacts on academics, social relationships and mental health are not yet clear. Moreover, we do not have a thorough understanding as to the coping strategies students are implementing to deal with the stress of COVID-19. To this end, the current study examined how the COVID-19 pandemic impacted university student academics, social relationships, and well-being, as well as the nature and extent of coping strategies endorsed by students to deal with the stress of the pandemic. Given the recent evidence that a subset of young people have increased their alcohol, tobacco and cannabis use during the COVID-19 pandemic (25, 26), we were particularly interested in exploring coping strategies pertaining to drug and alcohol use. In addition, considering evidence that young females are particularly impacted by COVID-19 (13), we focused on identifying gender differences in each of these domains. We predicted that females would report that the COVID-19 pandemic more negatively impacted their academics, social isolation, stress and mental health compared to males. We also predicted that students would employ a range of unhealthy coping strategies to deal with the stress of the pandemic, such as the use of alcohol and cannabis. Moreover, we hypothesized that unhealthy coping strategies, in particular substance use and eating fast food and/or sweets, would relate more strongly to greater negative impacts of the COVID-19 pandemic on stress and mental health. To conduct this project, we surveyed undergraduate students registered in academic classes throughout the summer (May–August) 2020 term. Ultimately, we hope that by identifying the specific challenges and stressors students are encountering during the COVID-19 pandemic, and how students are coping with these challenges, we may be better able to provide tailored supports and resources for these students.

## Methods

### Participants

This study comprised 366 undergraduate students from Carleton University (*M*_age_ = 21.0 years, range = 18–29 years). The majority of participants reported their gender identity as female (71.0%, *n* = 260), followed by male (28.1%, *n* = 103), gender queer (0.5%, *n* = 2), and transwoman (0.3%, *n* = 1). The sample was of mixed ethnic/racial backgrounds including participants who identified as Asian (12.3%, *n* = 45), South Asian (9.3%, *n* = 34), South East Asian (2.7%, *n* = 10), Arab/West Asian (7.4%, *n* = 27), Black (8.7%, *n* = 32), Latin American/Hispanic (3.3%, *n* = 12), Indigenous (0.8%, *n* = 3), White/European (49.4%, *n* = 181), and other (6.0%, *n* = 22).

When asked about their current employment, just under half of participants, 44.9% (*n* = 164) were unemployed, whereas 31.0% (*n* = 113) of participants reported being employed part-time, 16.7% (*n* = 61) were employed full-time, 7.4% (*n* = 27) reported their employment status as other and one participant did not answer this question. Among those who were unemployed, the majority (66.5%, *n* = 109) reported that their unemployment was due to the COVID-19 pandemic. Most participants responded that they currently reside in Canada, with 93.4% (*n* = 342) in Ontario, 1.4% (*n* = 5) in Alberta, 1.9% (*n* = 7) in Quebec, 0.5% (*n* = 2) in British Columbia, and 0.3% (*n* = 1) in each of Nova Scotia and Newfoundland & Labrador. Otherwise, 1.1% (*n* = 4) reported residing in China, and 0.3% (*n* = 1) reported residing in each of France, Bermuda, Anguilla, and the United States of America. When asked about living arrangements, 66.9% (*n* = 245) reported living in a household of 2–4 people, 23.2% (*n* = 85) reported living in a household of 5–8 people, 8.5% (*n* = 31) reported living alone, and 1.4% (*n* = 5) reported other living arrangements. Approximately a third of participants, 35.5% (*n* = 130), reported that their living arrangement had changed due to the COVID-19 pandemic.

### Procedure

Participants comprised undergraduate students from Carleton University, Ottawa, Ontario, Canada, recruited through the university's online research system. Participants completed this study during the COVID-19 pandemic, between May and August 2020. Within the context of the COVID-19 pandemic, in Ontario, a state of emergency was in effect from March 17, 2020 to July 24, 2020. This resulted in the closure of all schools, child care, indoor recreation facilities, restaurants, bars, and all non-essential services and businesses. From May through to August 2020, Ontario instituted a three-stage plan to lift economic restrictions, however, social distancing and social restriction guidelines remained, including restrictions on the size of gatherings. Thus, over the course of this study recruitment from Carleton University, classes were all being offered on-line, campuses were closed, and many social restrictions were in place.

Following informed consent, participants completed an online survey hosted on Qualtrics (Qualtrics, Provo, UT). The survey contained demographic questions (e.g., age, gender, mental health status) and questions related to the COVID-19 pandemic. Once the questionnaires were completed, all participants received an online debriefing form and were compensated with course credit. This study was cleared by the Carleton University Research Ethics Board (REB # 111775).

### Measures

#### Negative Impacts of the COVID-19 Pandemic

To assess the specific impacts of the COVID-19 pandemic, participants were asked separate questions regarding the extent to which the COVID-19 pandemic had negatively impacted their (1) mental health, (2) stress levels, (3) social relationships and (4) academic performance, with response options ranging from (0: not at all, 1: a little, 2: a moderate amount, 3: very much, 4: an extreme amount). Due to small *n*/cell for specific groups, responses were collapsed for statistical analyses into three categories: (0) not at all/a little, (1) a moderate amount, and (2) very much/an extreme amount. In addition to the above questions, participants were also asked to rate the difficulty of the social isolation caused by the COVID-19 pandemic, and the transition to online learning, with response items ranging from (0: very easy, 1: easy, 2: neutral, 3: difficult, 4: very difficult). For analyses, once again responses were collapsed into three categories: (0) very easy/easy, (1) neutral, and (2) difficult/very difficult.

#### Coping With the COVID-19 Pandemic

It was also of interest to ask participants how they had been coping with the COVID-19 pandemic. Participants were asked how often they had been employing a range of coping methods to deal with the stress of the COVID-19 pandemic with response options ranging from 0: not at all, 1: a little, 2: a moderate amount, 3: very much, 4: an extreme amount. For analyses, this scale was collapsed into three categories: (0) not at all/a little, (1) a moderate amount, and (2) very much/an extreme amount. The specific coping strategies assessed and asked to participants comprised separate questions regarding: (1) social media; (2) connecting with family/friends through videoconferencing (Zoom, FaceTime, etc.); (3) exercising; (4) sleeping; (5) eating fast food/sweets; and (6) using substances (vaping nicotine, alcohol and cannabis use). These options were selected based on common behaviors and coping strategies endorsed by young adults (22, 27–29).

### Statistical Analyses

Statistical analyses were performed using SPSS for Windows 27.0 (SPSS, Chicago, Illinois, USA). Prior to analyses, a number of validity checks were performed to ensure quality of data. These included the time to complete the survey and that responses on reverse coded items of scales aligned. There were too few individuals who reported their gender identity other than male/female (*n* = 3), thus, for gender-based analyses these individuals were excluded, however they were included in all other results. As data were grouped in ordinal categories [i.e., questions assessing the negative impacts of COVID-19 comprised three groups: (0) very easy/easy, (1) neutral, and (2) difficult/very difficult and questions assessing coping with COVID-19 comprised three groups: (0) not at all/a little, (1) a moderate amount, and (2) very much/an extreme amount], chi-square analyses were performed when assessing these questions according to gender. Due to the ordinal data, Spearman's Rho was used to correlate the responses to questions assessing the negative impacts of COVID-19 and the responses to the coping with COVID-19 questions. Significance was determined at *p* < 0.05.

## Results

### The Impacts of the COVID-19 Pandemic on Academic Outcomes

**Figure 1:**
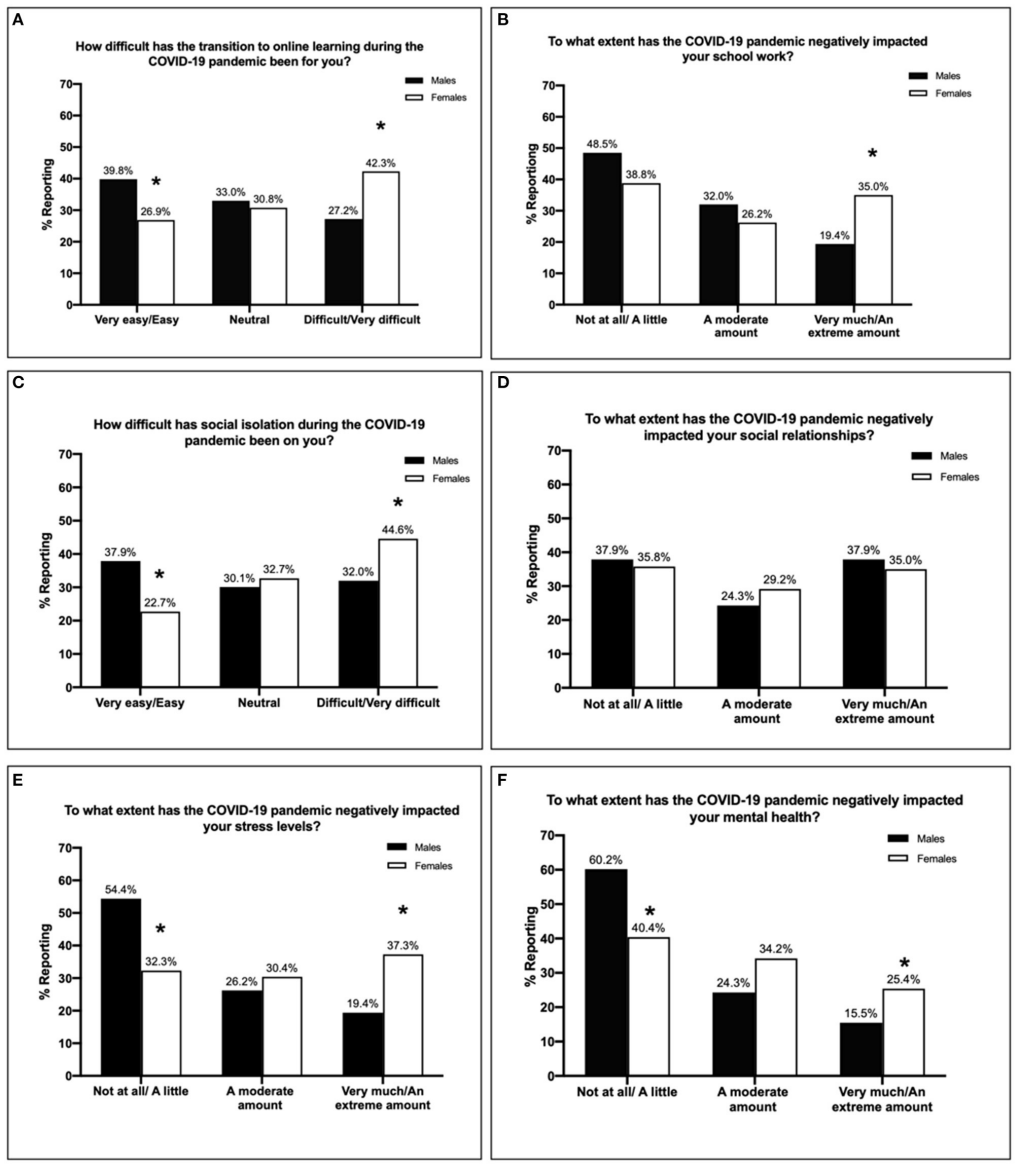
The difficulty of the transition to online learning during the COVID-19 pandemic **(A)**, and the extent of negative impact of COVID-19 on schoolwork **(B)**, according to gender. The difficulty of social isolation during the COVID-19 pandemic **(C)** and the extent of the negative impact of COVID-19 on social relationships **(D)**, according to gender. The negative impacts of COVID-19 on stress **(E)** and mental health **(F)**, according to gender. *p < 0.05 relative to males within the same category.

Of all participants, 37.7% (*n* = 138) reported that the transition to online learning during the COVID-19 pandemic was difficult or very difficult, whereas 31.1% (*n* = 114) reported being neutral, and 31.1% (*n* = 114) said the transition was easy/very easy. As shown in Figure 1A, this differed significantly by gender [$\chi_{\left(2,\;\text{N}=363\right)}^{2}$ = 8.56, *p* = 0.014]. Specifically, a greater proportion of females reported that the transition to online learning was difficult or very difficult compared to males (*p* < 0.05). In contrast, more males reported the transition was very easy/easy (*p* < 0.05) compared to females.

When asked about the extent of negative impact of the COVID-19 pandemic on schoolwork, 30.6% (*n* = 112) of participants reported that it negatively impacted them very much or an extreme amount, whereas 27.9% (*n* = 102) reported a moderate negative impact and 41.5% (*n* = 152) reported little to no negative impacts. This differed significantly by gender [$\chi_{\left(2,\;\text{N}=363\right)}^{2}$ = 8.44, *p* = 0.015; Figure 1B]. Again, a greater proportion of females reported that the COVID-19 pandemic had negatively impacted their schoolwork very much or an extreme amount compared to males (*p* < 0.05).

### The Impacts of the COVID-19 Pandemic on Social Isolation and Relationships

Of participants, 41.3% (*n* = 151) reported that social isolation had been difficult or very difficult, whereas 31.7% (*n* = 116) reported being neutral, and 27.0% (*n* = 99) said isolation was easy/very easy. This differed significantly by gender [$\chi_{\left(2,\;\text{N}=363\right)}^{2}$ = 9.29, *p* = 0.01]. As shown in Figure 1C, females were more likely to report social isolation as being difficult or very difficult compared to males (*p* < 0.05), whereas males were more likely to report social isolation as being easy or very easy compared to females (*p* < 0.05).

With regard to social relationships, 35.8% (*n* = 131) of participants reported that COVID-19 negatively impacted them very much or an extreme amount, whereas 27.9% (*n* = 102) reported a moderate negative impact and 36.3% (*n* = 133) reported little to no negative impacts. These negative impacts did not differ according to gender [$\chi_{\left(2,\;N=363\right)}^{2}$ = 0.91, *p* = 0.63; Figure 1D].

### The Impacts of the COVID-19 Pandemic on Stress and Mental Health

Of participants, 32.5% (*n* = 119) reported that COVID-19 negatively impacted their stress levels very much or an extreme amount, whereas 29.0% (*n* = 106) reported a moderate negative impact and 38.5% (*n* = 141) reported little to no negative impacts. As shown in Figure 1E, this differed significantly according to gender [$\chi_{\left(2,\;\text{N}=363\right)}^{2}$ = 17.08, *p* = 0.0002]. Namely, females were more likely to report the negative impacts of COVID-19 on stress levels to be very much or an extreme amount compared to males (*p* < 0.05), whereas males were more likely to report the negative impacts to be not at all or a little compared to females (*p* < 0.05).

Of participants, 23.0% (*n* = 84) reported that COVID-19 negatively impacted their mental health very much or an extreme amount, whereas 31.1% (*n* = 114) reported a moderate negative impact and 45.9% (*n* = 168) reported little to no negative impacts. These negative impacts differed significantly according to gender [$\chi_{\left(2,\;\text{N}=363\right)}^{2}$ = 11.79, *p* = 0.003]. Again, as shown in Figure 1F, females were more likely to report the extent of the negative impact on mental health to be very much or an extreme amount compared to males (*p* < 0.05). Once again, males were more likely to report that COVID-19 negatively impacted their mental health not at all or a little compared to females (*p* < 0.05).

### Coping With the COVID-19 Pandemic

Tables 1, 2 reflect how often participants endorsed specific coping methods to deal with the stress of the COVID-19 pandemic. As shown in Table 1, the majority of participants reported connecting with friends/family members through Facetime, Zoom, Skype, etc. (60.4%), and using social media (79.2%) as a coping mechanism at least a moderate amount. However, gender differences were found in relation to using these video-chat platforms, [$\chi_{\left(2,\;\text{N}=363\right)}^{2}$ = 16.36, *p* = 0.0003], and in relation to social media use, [$\chi_{\left(2,\;\text{N}=363\right)}^{2}$ = 14.86, *p* = 0.001]. Specifically, females were more likely to report using online technologies/applications very much or an extreme amount to cope with the stress of the COVID-19 pandemic compared to males (*p* < 0.05; Table 1).

**Table 1:** Percentage of coping methods endorsed to deal with the stress of the COVID-19 pandemic.

	**Not at all/a little % (*n*)**	**A moderate amount % (*n*)**	**Very much/an extreme amount % (*n*)**
**Connecting with friends/family through videoconference** (*n* = 366)	39.6 (*n* = 145)	29.8 (*n* = 109)	30.6 (*n* = 112)
Males (*n* = 103)	55.3 (*n* = 57)	25.2 (*n* = 26)	19.4 (*n* = 20)
Females (*n* = 260)	33.1 (*n* = 86)	31.5 (*n* = 82)	35.4 (*n* = 92)^*^
**On social media** (*n* = 366)	20.8 (*n* = 76)	30.3 (*n* = 111)	48.9 (*n* = 179)
Males (*n* = 103)	31.1 (*n* = 32)	35.0 (*n* = 36)	34.0 (*n* = 35)
Females (*n* = 260)	16.9 (*n* = 44)	28.1 (*n* = 73)	55.0 (*n* = 143)^*^
**Exercising** (*n* =366)	51.9 (*n* = 190)	27.3 (*n* = 100)	20.8 (*n* = 76)
Males (*n* = 103)	51.5 (*n* = 53)	24.3 (*n* = 25)	24.3 (*n* = 25)
Females (*n* = 260)	51.5 (*n* = 134)	28.8 (*n* = 75)	19.6 (*n* = 51)
**Sleeping** (*n* = 366)	24.0 (*n* = 88)	35.2 (*n* = 129)	40.7 (*n* = 149)
Males (*n* = 103)	33.0 (*n* = 34)	43.7 (*n* = 45)	23.3 (*n* = 24)
Females (*n* = 260)	20.8 (*n* = 54)	32.3 (*n* = 84)	46.9 (*n* = 122)^*^
**Eating fast food/sweets** (*n* = 366)	40.7 (*n* = 149)	28.1 (*n* = 103)	31.1 (*n* = 114)
Males (*n* = 103)	51.5 (*n* = 53)	31.1 (*n* = 32)	17.5 (*n* = 18)
Females (*n* = 260)	36.9 (*n* = 96)	26.9 (*n* = 70)	36.2 (*n* = 94)^*^

* *p < 0.05 relative to males within the same category*.

**Table 2:** Percentage of coping methods endorsed to deal with the stress of the COVID-19 pandemic.

	**Not at all/a little % (*n*)**	**A moderate amount % (*n*)**	**Very much/an extreme amount % (*n*)**
**Vaping nicotine products** (*n* = 366)	90.7 (*n* = 332)	4.1 (*n* = 15)	5.2 (*n* = 19)
Males (*n* = 103)	86.4 (*n* = 89)	6.8 (*n* = 7)	6.8 (*n* = 7)
Females (*n* = 260)	92.3 (*n* = 240)	3.1 (*n* = 8)	4.6 (*n* = 12)
**Drinking alcohol** (*n* = 366)	76.2 (*n* = 279)	15.6 (*n* = 57)	8.2 (*n* = 30)
Males (*n* = 103)	76.7 (*n* = 79)	16.5 (*n* = 17)	6.8 (*n* = 7)
Females (*n* = 260)	76.5 (*n* = 199)	14.6 (*n* = 38)	8.8 (*n* = 23)
**Using cannabis** (*n* = 366)	82.5 (*n* = 302)	6.6 (*n* = 24)	10.9 (*n* = 40)
Males (*n* = 103)	82.5 (*n* = 85)	6.8 (*n* = 7)	10.7 (*n* = 11)
Females (*n* = 260)	83.1 (*n* = 216)	6.2 (*n* = 16)	10.8 (*n* = 28)

Overall, just under half of participants (48.1%) reported exercising to cope with COVID-19 at least a moderate amount (Table 1). This did not differ significantly by gender [$\chi_{\left(2,\;\text{N}=363\right)}^{2}$ = 1.32, *p* = 0.52]. Approximately 60% of participants reported eating fast food/sweets at least a moderate to cope with COVID-19 (Table 1), which did differ by gender [$\chi_{\left(2,\;\text{N}=363\right)}^{2}$ = 12.59, *p* = 0.002]. Specifically, females were more likely to report eating fast food/sweets very much or an extreme amount to cope with COVID-19 compared to males (*p* < 0.05; Table 1). Furthermore, when examining sleep, 75.9% reported sleeping at least a moderate amount to cope with COVID-19 (Table 1). This also differed according to gender [$\chi_{\left(2,\;\text{N}=363\right)}^{2}$ = 17.48, *p* = 0.0002], in which females were more likely to report sleeping very much or an extreme amount to cope with COVID-19 compared to males (*p* < 0.05; Table 1).

Some participants reported using substances specifically to cope with the stress of the COVID-19 pandemic (Table 2). Overall, 9.3% reported vaping nicotine products, 23.8% reported drinking alcohol, and 17.5% reported using cannabis at least a moderate amount as a coping mechanism to deal with the stress of COVID-19 (Table 2). Neither vaping nicotine products [$\chi_{\left(2,\;\text{N}=363\right)}^{2}$ = 3.42, *p* = 0.18], drinking alcohol [$\chi_{\left(2,\;\text{N}=363\right)}^{2}$ = 0.55, *p* = 0.76], nor using cannabis [$\chi_{\left(2,\;\text{N}=363\right)}^{2}$ = 0.05, *p* = 0.98], differed significantly by gender.

### Relationships Between COVID-19 Coping Strategies and Outcomes

It was also of interest to examine the relationship between the coping strategies employed to deal with the stress of COVID-19 and the negative impacts of COVID-19 on schoolwork, social relationships, stress levels and mental health according to gender. As shown in Table 3, increased sleeping and eating fast food/sweets to cope with COVID-19 was associated with greater negative impacts on schoolwork for both males and females (*p*'s < 0.01). For females only, increased coping through social media use was also related to negative impacts on schoolwork (*p* < 0.01), an effect not found for males (Table 3). Whereas, for males, coping with COVID-19 by using cannabis was associated with more negative impacts on schoolwork (*p* < 0.01; Table 3). Upon examining the negative impacts of COVID-19 on social relationships, coping through the use of social media, sleeping, and eating fast food/sweets was associated with greater negative impacts on social relationships for both males and females (*p*'s < 0.05).

**Table 3:** Spearman correlations between coping strategies and the negative impacts of COVID-19 on school work, social relationships, stress levels and mental health.

	**Video-conference**	**Social media**	**Sleeping**	**Exercise**	**Fast food /sweets**	**Cannabis use**	**Vaping nicotine**	**Alcohol use**
**Neg. impact on school work**								
Overall (*n* = 366)	0.19^**^	0.25^**^	0.24^**^	−0.09	0.31^**^	0.11^*^	0.09	0.17^**^
Males (*n* = 103)	0.20^*^	0.17	0.29^**^	−0.06	0.29^**^	0.30^**^	0.17	0.19
Females (*n* = 260)	0.14^*^	0.25^**^	0.20^**^	−0.11	0.30^**^	0.06	0.08	0.17^**^
**Neg. impact on social relationships**								
Overall (*n* = 366)	0.03	0.20^**^	0.18^**^	−0.05	0.20^**^	0.04	−0.02	0.05
Males (*n* = 103)	−0.00	0.22^*^	0.21^*^	−0.03	0.23^*^	0.16	0.05	0.15
Females (*n* = 260)	0.05	0.19^**^	0.18^**^	−0.06	0.19^**^	−0.00	−0.05	0.02
**Neg. impact on stress**								
Overall (*n* = 366)	0.10	0.31^**^	0.22^**^	−0.04	0.35^**^	0.12^*^	0.15^**^	0.21^**^
Males (*n* = 103)	0.04	0.23^*^	0.27^**^	0.01	0.45^**^	0.26^**^	0.34^**^	0.41^**^
Females (*n* = 260)	0.07	0.30^**^	0.14^*^	−0.04	0.27^**^	0.06	0.10	0.14^*^
**Neg. impact on mental health**								
Overall (*n* = 366)	0.06	0.31^**^	0.20^**^	−0.10	0.33^**^	0.16^**^	0.10	0.20^**^
Males (*n* = 103)	−0.10	0.28^**^	0.21^*^	−0.04	0.41^**^	0.28^**^	0.29^**^	0.35^**^
Females (*n* = 260)	0.07	0.29^**^	0.15^*^	0.11	0.27^**^	0.13^*^	0.05	0.15^*^

* *p < 0.05*,

** *p < 0.01*.

With regards to negative impacts of COVID-19 on stress levels, coping mechanisms such as social media use, sleeping, and eating fast food/sweets were all associated with greater negative impacts on stress levels for both genders (*p*'s < 0.05), although as seen in Table 3, the social media effect was stronger for females. While consuming alcohol was also significantly related to greater negative impacts on stress levels for both genders (*p* < 0.05), the effect was much stronger for males compared to females (Table 3). Moreover, using cannabis and vaping nicotine to cope were also related to greater negative impacts on stress levels, but for males only (*p*'s < 0.01). Concerning negative impacts on mental health, coping mechanisms such as social media use, sleeping, eating fast food/sweets more frequently were related to greater negative impacts on mental health for both genders (*p*'s < 0.05). When it came to coping through the use of substances, cannabis use and alcohol use were associated with negative impacts on mental health for both males (*p*'s < 0.01) and females (*p*'s < 0.05), however, these effects were stronger for males (Table 3). Additionally, for males only, coping through vaping nicotine more frequently was related to greater negative impacts on mental health. Moreover, as shown in Table 3, exercise was not related to any outcomes of interest.

## Discussion

The COVID-19 pandemic, including its associated social restrictions, has certainly been a challenging situation for many individuals. Yet, it has become clear that not everyone has been equally affected by the pandemic. Among several disproportionally affected populations, young and emerging adults are particularly struggling with the current pandemic circumstances. Emerging adulthood is already a transitional and stressful period, filled with instability owing to changes in education, living arrangements, and relationships (30). This period corresponds to biological and developmental changes (31) and the onset of mental health disorders such as anxiety and depression (19). Moreover, emerging adulthood coincides with beginning postsecondary education, such as university. To date, much less is known about how the continuously evolving COVID-19 pandemic has impacted post-secondary students. However, if this group is already encountering a number of stress and mental health difficulties, the stress of the pandemic might further exacerbate these concerns. To this end, the current study investigated the impact of the COVID-19 pandemic on post-secondary students' academic experience, social relationships, feelings of isolation, and mental health. In addition, we examined the extent to which students were using several types of coping strategies to deal with different aspects of the pandemic.

Interestingly, we found that across all outcomes measured, the magnitude of impact of the COVID-19 pandemic varied across students. Specifically, roughly one third of students reported that the pandemic, and accompanying challenges, had little-to-no impact on academic performance, stress and mental health, and about one third indicated that COVID-19 had a moderate impact on these outcomes. These findings are encouraging as they suggest that many post-secondary students have not been considerably impacted by the pandemic. However, about one third of students in the current study indicated that the COVID-19 pandemic has been very (or extremely) difficult. In fact, the present data indicate that the pandemic has been particularly difficult on female students, a finding that parallels observations made in the general population (3, 14).

### Academic Outcomes

In response to the pandemic, many Universities abruptly suspended face-to-face learning in favor of online learning. This decision, while necessary, had the potential to lead to a variety of negative social, psychological, and academic consequences for post-secondary students (32). Indeed, our data reveal that more than one third of students in the current study reported that the transition to online learning was difficult or very difficult. Moreover, just under one third reported that the pandemic negatively impacted their schoolwork very much or an extreme amount. These data are in-line with recent reports that COVID-19 has had a substantial negative influence on the academic experiences of post-secondary students (24, 33). Specifically, students have reported that the experience of online learning has resulted in significantly higher levels of stress and isolation as well as negative mood, and significantly lower levels of relatedness, concentration, focus, motivation, and performance compared to traditional face-to-face learning (24). The current study also shows that female students were more likely than male students to report that the transition to on-line learning was difficult and that the COVID-19 pandemic has negatively impacted their schoolwork. Struggling academically may increase already elevated distress among the post-secondary population (34).

### Social Isolation and Relationships

Many sectors of the general population have shown a rise in self-reported loneliness during the pandemic as a consequence of social distancing measures and quarantines (35). Interestingly, the increased feelings of loneliness are greatest among younger populations (5, 6). In the current study, 41.3% of the students reported that social isolation due to COVID-19 was difficult or very difficult, and this was further differentiated according to gender, such that more female students (44.6%) reported social isolation being difficult/very difficult compared to males (32.0%). This is consistent with emerging literature showing that young adult females have experienced greater changes in levels of loneliness (36), and that female students are more likely to experience negative mental health consequences as a result of pandemic-induced changes in their social networks (16). This is of particular concern, based on previous reports that females tend to be more susceptible to the effects of loneliness on mental health (13, 37, 38). Moreover, while approximately one third of students in our study reported negative impacts of COVID-19 on their social relationships, this result did not differ by gender. It therefore appears that while female students are struggling with more feelings of isolation, both genders are equally struggling with their social relationships. This is perhaps not surprising, given the physical distancing measures and restrictions on social gatherings put in place by public health officials to mitigate the spread of COVID-19. However, these data are worrisome considering evidence that social support buffers against the negative effects of stressors (39).

### Stress and Mental Health

In the current study, one third of participants (32.5%) reported that the pandemic negatively impacted their stress levels very much or an extreme amount, and 23% reported similar negative impacts on their mental health. These data were expected given that the COVID-19 pandemic has been accompanied by many factors known to increase stress and emotional distress (40, 41), such as isolation from friends and family, loss of employment and income, unfamiliar public health measures, and uncertainty about the future (42). In line with these data, high rates of negative mental health outcomes and emotional distress as a consequence of the pandemic have also been found in the general population (2, 43, 44). However, what is most interesting is that, once again, the negative mental health consequences of the pandemic have been especially pronounced among younger populations (4). Our data support these findings by demonstrating that a proportion of university students are experiencing increased pandemic-related stress and mental health concerns. In addition, we found that the negative impacts of COVID-19 on stress levels and mental health were much more pronounced among female students (37.3 and 25.4%, respectively) compared to males (19.4 and 15.5%, respectively). While not specific to student populations, emerging literature similarly finds that young adult females demonstrate higher levels of depression, anxiety, and stress in the wake of COVID-19 (13, 14). Indeed, females more generally have experienced a greater elevation in levels of depression, emotional distress, and panic as a result of the pandemic (45).

### Coping With the COVID-19 Pandemic

A large body of research indicates that individuals not only differ in how they respond to, or cope with stressful experiences, but that differences in coping play an important role in determining various mental and physical health outcomes (46, 47). Thus, the selection of coping methods to contend with the stressor can regulate emotional responses (48). In this respect, problem-oriented coping methods (e.g., problem-solving) tend to be associated with positive outcomes, whereas emotion-focused coping strategies, such as rumination, blame and avoidance are often linked to negative outcomes (e.g., symptoms of depression) (47). Moreover, social support, which can serve as both a problem- and emotion-focused coping strategy, has frequently been shown to buffer the effects of stress and promote positive mental health outcomes (49). In the present study, we found that the frequency in use of *connecting with friends/family through videoconference* to cope with the stress of the pandemic was variable - some students reported using this method of coping very frequently, whereas others said that they video conferenced only a little bit if at all. However, video conferencing was more commonly used among female students. Connecting through video conferencing was unrelated to the social, stress and mental health outcomes measured, which may suggest that connecting through this platform might not provide the same benefit as connecting in-person. While use of the internet is undoubtedly useful and often necessary for communication in this new online era, the relationship between social media use and overall well-being is complex (50). In the context of the pandemic, social media offers a wealth of information, so much so that the World Health Organization (WHO) developed the term “infodemic” to describe the overabundance of information available online (as well as offline), and argue that this infodemic and associated misinformation can be harmful to people's physical and psychological health (51). That said, social media can also work to maintain social networks and a sense of normalcy (52), which holds the potential to be beneficial in attempting to cope with the pandemic.

Social media use, as a method of coping with the pandemic was very common in the present study, especially among female students. To be sure, these data are in line with findings from a non-student sample where females were more likely to cope with the social isolation of COVID-19 through social media use than males (53). More importantly, however, we found that frequent use of social media was associated with a greater negative impact on schoolwork and perceived stress levels among females. In contrast, among all participants, frequent social media use similarly related to negative mental health. Indeed, several recent reports link social media use to poorer mental health during the COVID-19 pandemic (9, 54, 55). The relationship between frequent, or excessive, social media use and poor mental health has been shown prior to the pandemic (56, 57). In this regard, excessive social media use and its impacts on mental and well-being, including risk of depression, anxiety and suicide, has been of concern for several years (50, 58, 59). However, most of the data on the topic has been correlational, making it difficult to attribute causality. On the one hand, for some individuals frequent social media use might lead to poor mental health. On the other hand, it is reasonable to think that some individuals who are experiencing depression and anxiety may be more likely to engage with social media, possibly as a method of support. Social media can have some beneficial purposes (60), including reducing stigma around mental health (61). In the context of the current COVID-19 pandemic, more research will be needed to determine whether different reasons for use of social media are predictive of different mental health outcomes.

In the present study, we found that increased frequency of sleep was generally associated with a greater negative impact on academic performance, social relationships, stress and mental health. Moreover, while students were typically sleeping more in response to the COVID-19 pandemic, this might not necessarily mean their sleep quality is good, as sleep disturbances have been reported during the pandemic (62, 63). Sleeping more often or for longer periods of time could also represent an avoidance style of coping, which has often been associated with poorer outcomes (64, 65). A notable proportion of post-secondary students in the current study also indicated that they were eating fast food and sweets to cope with the COVID-19 pandemic, and this was especially the case among female students. At first glance, this might seem like an odd or trivial observation. However, emotional eating, which is often characterized by the intake of high fat and high carbohydrate foods, is a common method of coping with stress, particularly among females, and is often associated with distress and psychopathology (66–68). In this respect, emotional eating reflects emotion-focused and avoidance coping (69), which have generally been viewed as ineffective coping strategies that often exacerbate stressful experiences over time (47). However, in this context, while females were more likely to use this method to cope, eating fast foods and sweets was linked to negative impacts on stress and mental health among both male and female students (Table 3).

When it came to using substances, 17.5% of participants reported endorsing substance use as a coping strategy to deal with the stress of the COVID-19 pandemic a moderate to extreme amount. While there were no gender differences in the frequency of using substances to cope with the pandemic, we did find an interesting relationship between coping with substances and academics. Specifically, coping through increased cannabis use was significantly related to greater negative impacts of COVID-19 on schoolwork among males, but not females. Furthermore, the negative impact of COVID-19 on stress and mental health was more strongly associated with increased coping through the use of cannabis, alcohol and vaping nicotine among males. Prior to the pandemic, studies had found that increased stress was associated with substance use among students (70). In fact, initiation or increase in substance use to cope with COVID-19 related stress has been most commonly reported by individuals aged 18–24 years (71). The gender difference in the current study is a noteworthy distinction to make, considering earlier literature showing that male students more frequently report coping with stress through substance use than do females (20). Further, evidence indicates that male students report higher rates of cannabis use than do females (72–74), putting them at greater risk for the development of a cannabis use disorder (75), and/or adverse academic outcomes (76).

### Limitations and Future Directions

There are some limitations that must be considered when interpreting the results of this study. The current study was comprised of self-report measures asking questions regarding the impacts of the COVID-19 pandemic. Thus, it is possible that current mood states could have impacted responses. To be sure, longitudinal assessments of COVID-19 are needed to examine the impacts of the COVID-19 pandemic, and how this changes over time. Additionally, it is worth nothing that our sample consisted of students who were enrolled in courses during the summer term. Students who are taking classes during COVID-19, and therefore eligible to participate research studies, may be those who are coping more effectively with the pandemic and experiencing less of a decline in well-being and/or have greater financial security. Nevertheless, we observed trends in our data that align with predictions from other studies related to mental health and coping among young adults, suggesting that our data may be representative of other university student populations. Finally, the current sample contained more female participants than males, and therefore some of the cells for our comparisons of coping strategies by gender were small. This was particularly apparent when assessing cannabis use and vaping by gender. For this reason, the current study could have benefited from a larger number of male participants.

Taken together, it is apparent that the COVID-19 pandemic is having a substantial impact on post-secondary students' academic experience, social relationships, and mental well-being. This is very problematic, as prior to the COVID-19 pandemic one in three university students had a mental health and/or substance use disorder (21), indicating that this is already an at-risk group. Female students in particular are reporting greater negative impacts and difficulties as a result of COVID-19. Related to these negative impacts, females are more likely to cope with the pandemic through the use of social media, potentially because they are struggling more with social isolation. By contrast, male students who are coping through the use of substances are reporting greater negative impacts on academics, stress, and mental health. In sum, these results emphasize the urgency and importance of developing support systems to mitigate the extensive negative impacts of the COVID-19 pandemic on the student population, and the development of interventions and treatments that are specific to the gendered impacts of the pandemic on mental health and well-being. Addressing student well-being and developing systems to mitigate potential declines in well-being will continue to be essential as the pandemic evolves.

## Data Availability Statement

The datasets presented in this article are not readily available because these data were not approved to be shared outside of the research team. Requests to access the datasets should be directed to robynmcquaid@cunet.carleton.ca.

## Ethics Statement

The studies involving human participants were reviewed and approved by Carleton University Research Ethics Board. The patients/participants provided their written informed consent to participate in this study.

## Author Contributions

KH, RM, RG, ZP, and AA contributed to the inception and design of the current experiment. Data collection were performed by RP and FS. Data analysis were performed by RP, FS, RG, and RM. The writing and editing of the manuscript was performed by RP, FS, AA, RG, KH, ZP, and RM. All authors contributed to the article and approved the submitted version.

## Conflict of Interest

The authors declare that the research was conducted in the absence of any commercial or financial relationships that could be construed as a potential conflict of interest.
